# A Comparison of Four Caries Risk Assessment Methods

**DOI:** 10.3389/froh.2021.656558

**Published:** 2021-04-28

**Authors:** John D. B. Featherstone, Yasmi O. Crystal, Pamela Alston, Benjamin W. Chaffee, Sophie Doméjean, Peter Rechmann, Ling Zhan, Francisco Ramos-Gomez

**Affiliations:** ^1^Department of Preventive and Restorative Dental Sciences, School of Dentistry, University of California, San Francisco, San Francisco, CA, United States; ^2^Pediatric Dentistry Department, College of Dentistry, New York University, New York, NY, United States; ^3^Comprehensive Pediatric Dentistry, Bound Brook, NJ, United States; ^4^Department of Orofacial Sciences, School of Dentistry, University of California, San Francisco, San Francisco, CA, United States; ^5^Department of Operative Dentistry and Endodontics, UFR d'Odontologie de Clermont-Ferrand, Clermont-Ferrand, France; ^6^EA 4847, Clermont-Ferrand, France; ^7^Université Clermont Auvergne, Clermont-Ferrand, France; ^8^Service d'Odontologie, CHU Estaing, Clermont-Ferrand, France; ^9^Section of Pediatric Dentistry, School of Dentistry, University of California, Los Angeles, Los Angeles, CA, United States

**Keywords:** caries management, caries risk assessment, dental caries, fluoride, infants and toddlers

## Abstract

**Introduction:** Caries risk assessment (CRA) is essential as the basis for successful management of dental caries. Of the many published CRA tools, four well-known ones are CAMBRA, Cariogram, American Dental Association (ADA), and American Academy of Pediatric Dentistry (AAPD) CRAs. The predictive accuracy of CAMBRA and Cariogram CRA tools have been examined in clinical outcomes studies in thousands of patients and the tools are widely used all over the world. The purpose of the present paper is three-fold, namely (1) to briefly review, compare and contrast these four CRA methods, (2) to provide a concise method for CRA introducing a quantitative component to the CAMBRA forms (CAMBRA 123), and (3) to guide the choice of CRA methods that will support caries management decisions.

**Comparison of Caries Risk Assessment Methods:** In the present evaluation, the above-mentioned four CRA methods for ages 0–6 years and 6 years-adult were compared using 26 hypothetical patients (13 per age group). Comparison results show that Cariogram and CAMBRA categorized patients into identical risk categories. Each of the ADA and AAPD tools gave different results than CAMBRA and Cariogram in several comparison examples. CAMBRA 123 gave the same caries risk level results as the Cariogram and the CAMBRA methods for all hypothetical patients for both age groups.

**Conclusions:** Both the Cariogram and the CAMBRA CRA methods are equally useful for identifying the future risk of dental caries. CAMBRA 123 shows promise as an easy-to-use quantitative method for CRA in clinical practice. The health care providers will be the ones to decide which CRA method will allow them to establish individualized, successful caries management therapies and how to combine these for the best care of their patients.

## Introduction

Dental caries continues to be a large problem for children and adults across the world [1]. Early childhood caries (ECC), when untreated, negatively affects the health, development and growth of children, and the well-being of their families [2, 3]. Severe cases of ECC are very difficult to manage and are often followed by future decay [4–6]. In a survey in the US in 2011–2012, among adults aged 20–64 years, 91% had experienced dental caries and 27% had untreated tooth decay [7]. Furthermore, children with caries in their primary dentition are three times more likely to develop caries in their permanent teeth [8]. Dental caries continues to be a major health problem.

**Figure 1:**
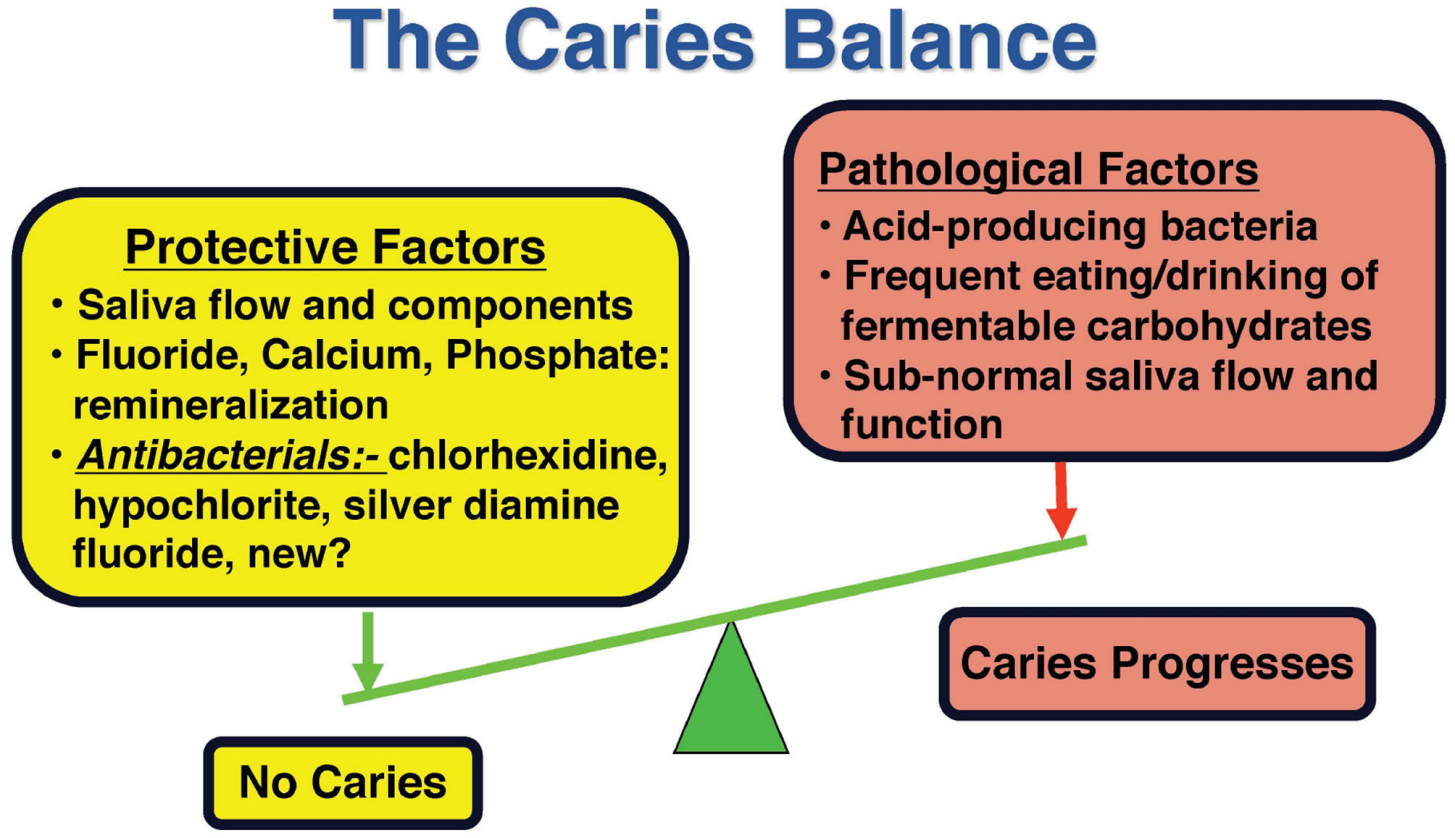
Schematic representation of the caries balance. Modified from Featherstone with permission [11].

It has become well-established that dental caries is a multifactorial disease involving numerous species of bacteria, dysbiosis of the biofilm on the teeth (dental plaque) driven by dietary habits that include frequent ingestion of fermentable carbohydrates, salivary dysfunction, inadequate preventive strategies and more [9, 10]. Its multifactorial nature, complicated by social determinants of health, does not allow for one preventive or therapeutic measure that is right for all patients. Dental caries can be simply described as a balance between caries pathological and preventive factors as illustrated in Figure 1 [9, 11]. The disease becomes apparent when pathological factors overcome the preventive factors resulting in breakdown of the dental tissues which can lead to pain and tooth loss. In young children aged 2–5 years in the US 75% of the 9 burden of caries occurs in ~8% of the population, and in older children aged 12–19 years 75% of caries occurs in 29% of the children [12]. These numbers emphasize the need for reliable caries risk assessment (CRA) and identification of those at high risk for future caries. Caries risk is the likelihood of the patient having new caries lesions (active white spots, non-cavitated approximal lesions, cavitated lesions) in the near future. The management of dental caries can be complicated, especially for patients with several pathologic caries risk factors. This is especially the case for patients with special needs.

The successful management of dental caries relies upon assessment of the caries risk of the individual and a treatment plan with personalized therapy that is derived from the details recorded in the risk assessment [13–18]. There are numerous published CRA methods and guidelines for caries management several of which are listed in Table 1 [19–48]. Assessment of the risk level for future occurrence of dental caries lesions is an important first step in managing dental caries and should be done periodically to monitor oral health changes over time.

**Table 1:** Caries risk assessment (CRA) tools-partial listing.

**CRA title**	**Source name (Reference)**
ADA	American Dental Association [19, 20]
AAPD	American Academy of Pediatric Dentistry [21]
AAPD-CAT (old version)	American Academy of Pediatric Dentistry [22]
AAP	American Academy of Pediatrics [23]
CAMBRA- “Caries Management by Risk Assessment”	University of California, San Francisco [24–28]
CDHCS-Domain #2 CRA Form	California Department of Health Care Services [29]
CariFree	CariFree, Oregon [30, 31]
CMS - Caries Management System	University of Sydney, NSW, Australia [32]
Cariogram (Internet-based Program)	Malmö University, Sweden [33]
DCRAM-Dundee Caries Risk Assessment Model	University of Dundee, Scotland [34]
EBH now-Online Search Engine for CRA	McGill University, Canada [35]
FDI	Fédération Dentaire Internationale-World Dental Federation [36]
MSB - My Smile Buddy (Electronic iPad based program)	Columbia University College of Dental Medicine [37, 38]
NUS - Caries Risk Assessment Tool	National University of Singapore [39]
PreViser electronic Caries Risk Assessment Tool	PreViser [40]
Texas Health and Human Services	Texas Health and Human Services [41]
UCC	University College Cork (Ireland) [42]

One of the questions that remains unanswered is which CRA tool (CRAT) to use and which CRATs are validated with clinical studies. There are several systematic reviews of CRATs and methods that have reported the “state of the art” over the past decade [13, 18, 49–53]. The earlier reviews came to similar conclusions that more studies are needed, especially to clinically validate the CRATs. Some of these reviews dealt only with CRATs for young children under the age of 6 years and others dealt with children of all ages and adults. Tellez et al. [50] concluded that “the validity of standardized CRA models still remains limited.” They also stated that “there is an urgent need to develop valid and reliable methods for caries risk assessment that are based on best evidence for prediction and disease management rather than opinions of experts.” Mejare et al. [13] concluded that the “accuracy of prediction models should be validated in at least one independent population.” Senneby et al. [51] wrote that “improved CRA methods are needed”. Christian et al. [53] summarized their review by stating “Studies on tools that were assessed as having strong evidence for content validity identified the relevant risk factors for caries in the population being studied, before developing and testing their respective CRATs.” They also suggested that the evidence to inform the selection of current CRATs for children is mostly yet to be established. The most recent systematic review by Schroth et al. [18] reported extensive assessment of each of the possible caries risk assessment components that contributed to numerous published CRATs. However, the review was done 4 years prior to the paper being published so their extensive tables did not include some of the most recent studies in the field. It is interesting to note that in their discussion these authors referred to recent reports that were published after their systematic review was completed and concluded that “CRA tools are not without limitations. Only a handful are validated (referencing specifically the Cariogram and CAMBRA tools).” They also stated a general observation relevant to several CRATs that “some of the combined factor questions are not evidence-based, developed by expert panels rather than based on systematic reviews of the literature.”

Concurrently with, and subsequent to these systematic reviews being conducted, both Cariogram and CAMBRA methods have been examined in several clinical studies that have not only demonstrated good caries risk assessment capabilities but also that caries management based upon risk assessment is successful clinically as reviewed in recent publications [14, 54, 55]. Several of the questions highlighted above have now been addressed. These results are described more fully below in the present review.

The present paper is not designed to be a systematic review, since many have already been published. Neither is it supposed to be a comprehensive review of available CRATs. Instead, it aims to provide an update on four specific CRATs that are readily available and used internationally, namely Cariogram, CAMBRA, AAPD and ADA (Table 1). This paper is designed as a direct head to head comparison of these four CRATs and to fill in some of the knowledge gaps identified above.

Therefore, the purpose of the present paper is three-fold, namely [1] to briefly review, compare and contrast four CRA methods, [2] to provide a concise method for CRA introducing a quantitative component to the CAMBRA forms (CAMBRA 123), and [3] to guide the choice of CRA methods that will support caries management decisions.

## Overview of Caries Risk Assessment Methods

Assessing a patient's baseline caries risk level can assist in predicting future occurrence of caries lesions and thereby better inform potential management and monitoring strategies to facilitate optimum oral health outcomes. A summary of systematic reviews of CRATs is provided in the introduction, together with the rationale for the present paper. As stated, the present paper aims to provide an update on four specific CRATs that are readily available and used internationally, namely Cariogram, CAMBRA, AAPD, and ADA.

In this publication the term “caries lesion” is used throughout to describe a dental lesion (cavitated or non-cavitated) caused by the dental caries process (“caries lesion” can also be referred to as a “carious lesion”). In addition, pathological and protective risk factors of dental caries can be biologic or environmental. In this publication environmental factors will include social determinants of health (like poverty, health literacy or access to care) that have shown predictive value for future disease. Successful management of dental caries requires a risk-based approach to formulate an individualized treatment plan using a chronic disease management model, which aims at targeting the risk factors (biological and environmental) that contribute to the establishment and progression of this multifactorial disease. This individualized treatment plan should include behavior/lifestyle modification (for diet improvement, less sugar intake and plaque control) and non-surgical caries management [15, 16, 27], in addition to appropriate minimally invasive restorative treatment if needed. The caries risk level determines the personalized caries management approach for each individual patient. Personalization further takes into consideration the behavioral barriers of the individual child or adult and the social context of the child/family/individual. The final determination of caries risk lies with the health care provider, based upon validated risk assessment guidelines coupled with other factors observed by the practitioner and his/her clinical judgment.

For decades there have been numerous attempts to provide methodology to predict future dental caries, to assess caries risk and to manage the disease process [56–59]. There are many publications related to these topics, including those for ages 0–6 years [15, 19, 21, 27, 54] as well as older children and adults.

As described above, numerous CRA tools/forms have been developed and published or are available online. A partial list is provided in Table 1 [19–48].

In this paper, we will provide an overview, compare and contrast four well-known published CRA methods from Table 1 for young children, older children and adults, including: (a) Cariogram (Malmö University, Sweden) [33], CAMBRA [University of California San Francisco (UCSF)] [24, 26–28], (c) American Dental Association (ADA, Chicago) [19, 20], and (d) American Association of Pediatric Dentistry (AAPD) [21].

### Cariogram Caries Risk Assessment for All Ages

The Cariogram method was developed over many years, primarily by personnel from the University of Malmö in Sweden [33, 54]. A computer application is available online that enables a caries risk assessment for patients of all ages based upon numerous clinical observations, preventive factors and risk factors that are entered into the program. This internet version was launched in 2004. An algorithm is used to calculate the percentage risk and classifies the patient as low, moderate or high risk. The method has been successfully assessed in several clinical studies [54, 60–63]. The instructions emphasize that the health care provider makes the final risk determination based upon the Cariogram procedure and a personal knowledge of the patient. There is provision in the Cariogram for insertion of a clinical opinion.

### CAMBRA Caries Risk Assessment for Ages 0–6 Years and 6 Years Through Adult

The CAMBRA (caries management by risk assessment) CRA tool was developed over decades by personnel at the UCSF, is based upon research on key factors that contribute to caries progression or reversal. The tool was launched in the clinics at UCSF in 2003 and has been updated since then based upon clinical outcomes [24–26, 28, 46]. It provides a CRA form for two age ranges, namely ages 0–6 years and 6 years through adult. The caries risk level is determined by the health care provider as low, moderate, high or very high/extreme by following the instructions and visualizing the “caries balance” [26, 28] to weigh the clinical observations, preventive factors, biological and environmental risk factors and finally the clinical judgment of the care provider as described in Tables 2, 3 and earlier publications [24–26, 28]. Tables 2, 3 are CAMBRA risk assessment forms for the two age groups, updated from previous publications [26, 28] to provide a better flow for clinical practice and more clarity for the end user. The forms continue to utilize the previous evidence-based key factors for caries risk assessment. A more detailed description of how CAMBRA CRAs are used in clinical practice is provided elsewhere [26, 28].

**Table 2 (Part 1):**
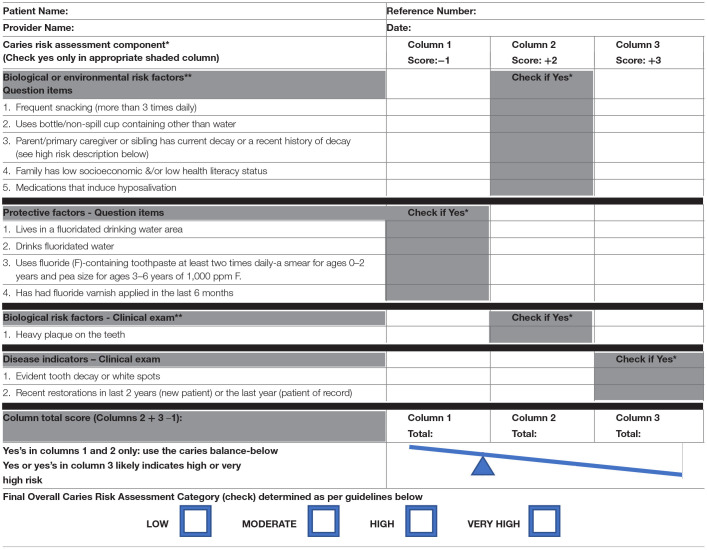
Updated CAMBRA caries risk assessment form^#^ for ages 0–6 years (January 2021)^##^.

*Check only the yes answers in the appropriate shaded column. Enter the score of −1, +2 or +3 for each yes checked. Unshaded columns are left blank. Assess the caries risk as per instructions in Table 2 (part 2) below.

**Biological and environmental risk factors are split into (a) question items, (b) clinical exam.

# *Modified from Featherstone et al. [26] with permission of California Dental Association Journal*.

## *This material may be used free of charge for the purposes of patient care, education, academic works, research, health promotion, health policy and related activities. However, permission must be obtained before this material is used for commercial purposes*.

*Refer to the second page of this form (part 2) for instructions for use as guidelines for caries risk assessment*.

**Table 2 (Part 2):** Caries risk assessment guidelines 0–6 years.

**The dental caregiver has the responsibility of making a caries risk assessment and then deciding on a caries management plan for the patient that leads from the risk assessment and a personalized assessment of the needs of the individual patient. These guidelines for assessing the risk as low, moderate, high or very high can assist in the process.** 1. **Low risk**. If there are protective factors, very few or no risk factors, no disease indicators, and the protective factors prevail, the patient is at low risk. 2. **Moderate risk**. If there are no disease indicators and the risk factors and protective factors appear to be balanced then a moderate caries risk determination is appropriate. If in doubt move the moderate to a high classification. 3. **High risk**. If there is a “YES” in column 3 (one or both disease indicators) the patient is very likely at high risk. Even if there are no “yes” disease indicators the patient can still be at high risk if the risk factors definitively outweigh the protective factors. Parent or caregiver with current or recent dental decay most likely indicates high caries risk for the child. 4. **Very high risk**. If the above process indicates high risk and the existing or recent decay is severe and/or extensive a designation of “very high” caries risk is appropriate and will guide a more aggressive caries management plan. Any items checked “yes” should also be used as topics to modify behavior or determine additional therapy.

**Use the following modified caries balance** to visualize the overall result and determine the risk level. It may be helpful to allocate scores for each “yes” checked on the risk assessment form with a score of−1 for yes's in column 1, and +2 and +3 respectively for yes's in columns 2 and 3. The final total will help guide the risk level decision. **Low** = −4 to −1; **Moderate** = 0 to +3; **High** = +4 to +13; **Very high** = +14 to +18 and/or is a high risk level plus extensive and/or severe recent or existing decay.

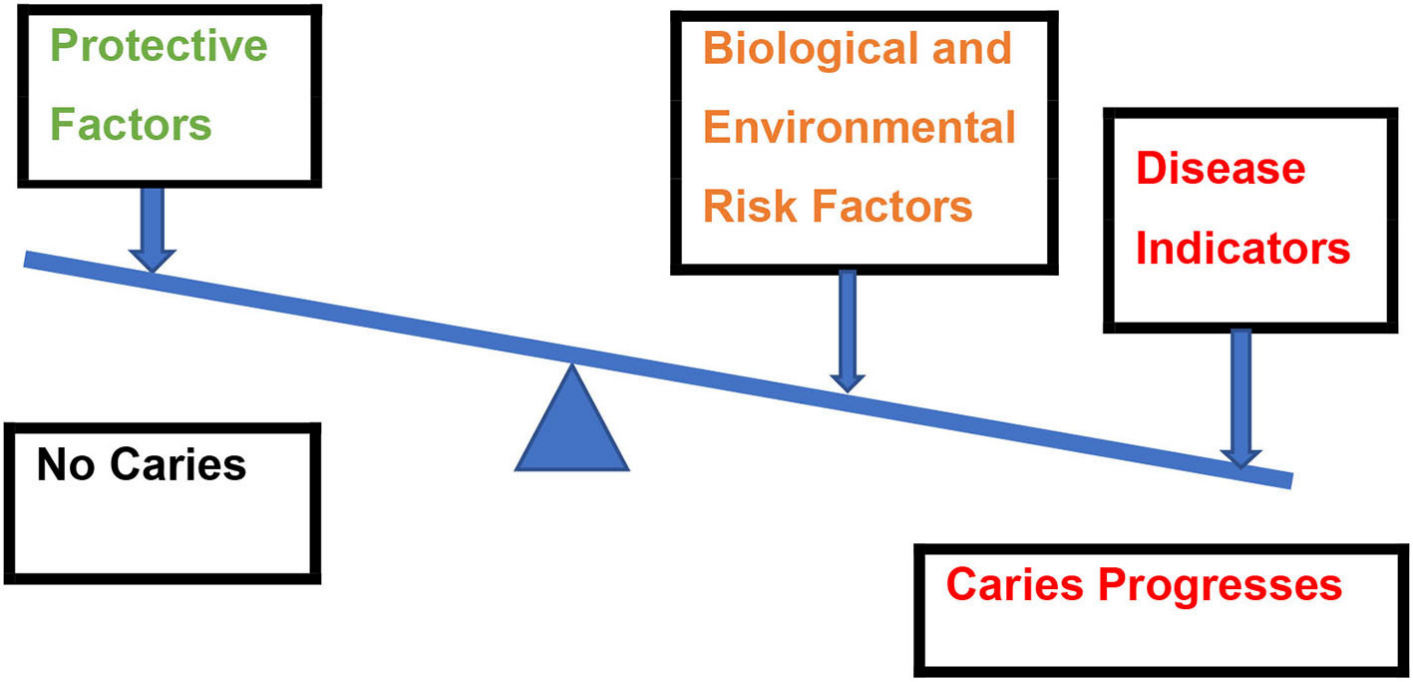

**Additional caries-related components for caries management and caregiver/patient counseling.Record in patient chart at each visit**.
Dietary counseling to reduce frequency and amount of fermentable carbohydrates, especially sucrose, fructose (high fructose corn syrup) and continual fruit juice (e.g., apple juice). Record number and type of daily snacks, drinks and juices used.
Bottle used continually, bottle used in bed or nursing on demand. Record details provided.
Fluoride (F) toothpaste use. Note frequency and amount used at each visit.
Record all recommended therapy such as F toothpaste, F varnish, use of silver diamine fluoride in appropriate cases. Record usage provided by parent/caregiver.
Record medications at each visit and check for changes.
Record participation in assistance programs such as “school lunches,” “head start,” appropriate to the state or country.
Child has developmental problems/child has special care needs (CHSCN).
Inadequate saliva flow and related medications, medical conditions, or illnesses.
**Discuss self-management goals with parent/caregiver and set two goals together at each visit. Provide in writing**.

**Table 3 (Part 1):**
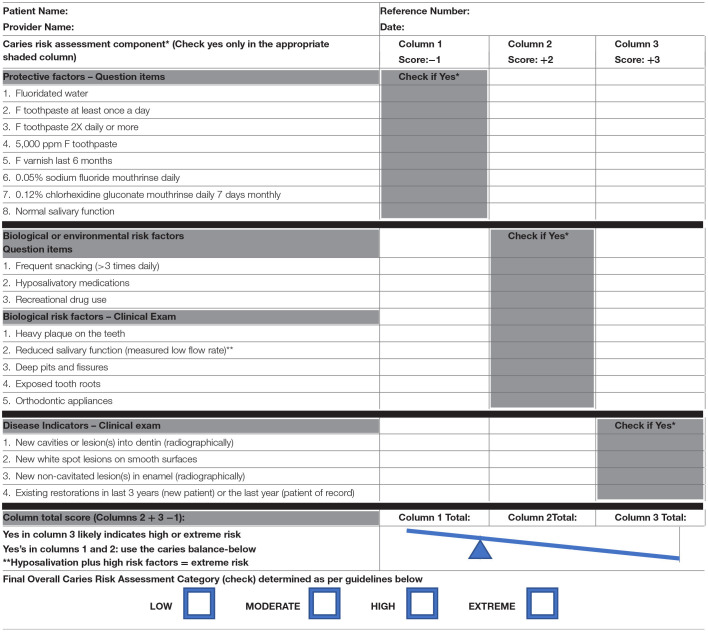
Updated CAMBRA caries risk assessment form^#^ for ages 6 year through adult (January 2021)^##^.

*Check only the yes answers in the appropriate shaded column. Enter a score of −1, +2 or +3 for each yes checked. Unshaded columns are left blank. Assess the caries risk as per instructions in Table 3 (part 2) below.

# *Modified from Featherstone et al. [28] with permission of California Dental Association Journal*.

## *This material may be used free of charge for the purposes of patient care, education, academic works, research, health promotion, health policy and related activities. However, permission must be obtained before this material is used for commercial purposes*.

*Refer to the second page (part 2) for instructions for use as guidelines for caries risk assessment*.

**Table 3 (Part 2):** Caries risk assessment guidelines for ages 6 years through adult.

**The dental caregiver has the responsibility of making a caries risk assessment and then deciding on a caries management plan for the patient that leads from the risk assessment and a personalized assessment of the needs of the individual patient. These guidelines can assist in the process.** **Determining the caries risk as low, moderate, high or extreme - guiding principles** 1. **Low risk**. If there are no disease indicators, very few or no risk factors and the protective factors prevail, the patient is most likely at low risk. Usually this is obvious. 2. **Moderate risk**. If the patient is not obviously at high, or extreme risk and there is doubt about low risk, then the patient should be allocated to moderate risk and followed carefully, with additional chemical therapy added. An example would be a patient who had a root canal as a result of caries 4 years ago, and has no new clinical caries lesions, but has exposed tooth roots and only uses a fluoride toothpaste once a day. 3. **High and extreme risk**. One or more disease indicators most likely signals at least high risk. If there is also hyposalivation the patient is likely at extreme risk. Even if there are no positive disease indicators the patient can still be at high risk if the risk factors definitively outweigh the protective factors. Think of the caries balance: visualize the balance diagram as illustrated below. Any items checked “yes” should also be used as topics to modify behavior or determine additional therapy.

**Use the following modified caries balance** to visualize the overall result and determine the risk level. It may be helpful to allocate scores for each “yes” checked on the risk assessment form with a score of −1 for yes's in column 1, and +2 and +3, respectively, for yes's in columns 2 and 3. The final total will help guide the risk level decision. **Low** = −8 to −2; **Moderate** = −1 to +2; **High** = +3 to +17; **Extreme** = +18 to +30 and/or is a high risk level plus measured or observed hyposalivation. Use the caries balance to visualize the overall result and determine the risk level for the individual patient.

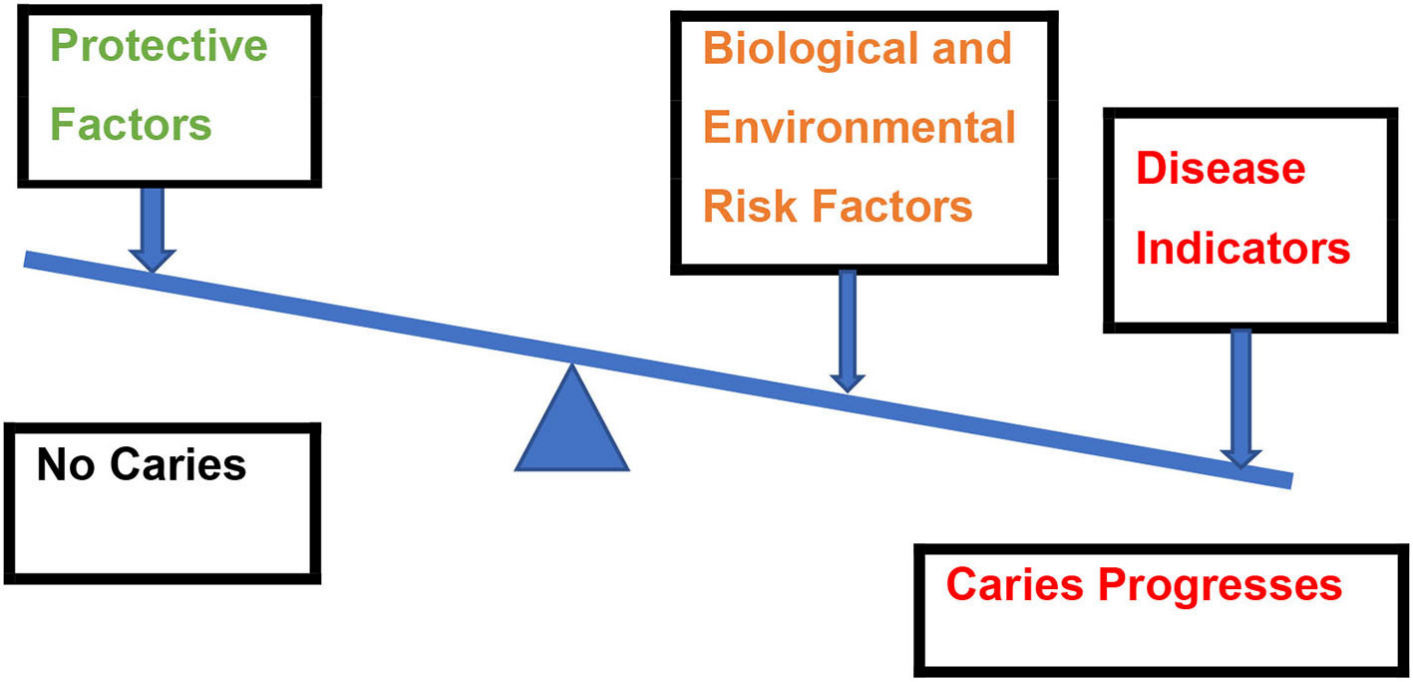

**Additional caries-related components for caries management and caregiver/patient counseling. Record in patient chart at each visit**.
Dietary counseling to reduce frequency and amount of fermentable carbohydrates. Record number and type of daily snacks, drinks and juices used.
Oral hygiene and fluoride (F) toothpaste use. At each visit note frequency and amount used.
Record all recommended therapy such as F toothpaste, F varnish, chlorhexidine and usage by patient.
Record medications at each visit and check for changes.
Record participation in assistance programs such as “school lunches,” “head start,” appropriate to the state or country.
Child or adult has developmental problems or special care needs (CHSCN).
Inadequate saliva flow and related medications, medical conditions, or illnesses.
**Discuss self-management goals with caregiver/patient and set two goals together at each visit. Provide in writing**.

The CAMBRA CRA tool has been shown to be highly predictive of future caries in three different clinical outcomes studies, totalling more than 20,000 patients, for the age group 6 years through adult and for the age group 0–5 years [55, 64–66]. Details of the evidence for the clinical success have been summarized and published previously [26, 28, 55, 64–66].

### ADA CRA Forms

The American Dental Association has published CRA forms for ages 0–6 years and >6 years of age [19, 20]. These forms were developed by expert panels. Each of the forms contain clinical observations, preventive factors and risk factors arranged into three columns, namely low risk, moderate risk and high risk. Patients are classified as having low, moderate or high caries risk by the health care provider depending on where the checked boxes on the form fall. To our knowledge there have been no published clinical outcomes studies that validate these forms and procedures even though they are widely used in the US and around the world.

### AAPD CRA Forms

Similar to the ADA, the American Academy of Pediatric Dentistry has published CRA forms for ages 0–5 years and 6 years of age or older [21]. These forms were developed by an expert panel and are updated from the original CAT (Caries Risk Assessment Tool) [22] of the AAPD. Each of the forms contains clinical observations, preventive factors and risk factors arranged into three columns, namely high risk, moderate risk and low risk. Patients are classified as low, moderate or high risk depending on where the checked boxes on the form fall and guidelines in the text. These forms and procedures are widely used for children in the US and around the world even though there is limited clinical outcomes information published.

#### Previous Comparisons of Caries Risk Assessment Tools

Very few head-to-head comparisons of CRATs have been published. Gao et al. [48] compared the Cariogram [33], CAMBRA [25], AAPD-CAT [22] and NUS (National University of Singapore) [39] CRAs in a clinical study predicting ECC in young pre-school children. They concluded “the algorithm-based programs (Cariogram and NUS) generated better predictions.” CAMBRA and AAPD-CAT CRAs that relied considerably on the judgment of the provider, gave excellent sensitivity but lower specificity. Since that study was completed both CAMBRA and AAPD-CAT have been updated [21, 26, 28]. The CAMBRA 0–5 CRA has also been validated in clinical outcomes studies [64, 65] and in this publication we are introducing a quantitative component to the CAMBRA forms (CAMBRA 123, see below).

A recent study by Agouropoulos et al. [67] using patient clinical outcomes compared the Cariogram CRA tool, the CAMBRA CRA 0–5 year method and the AAPD CRA 0–5 year tool in a prospective study over 2 years [67]. The Cariogram and CAMBRA methods behaved similarly with very good ROC (Receiver Operating Characteristic) curves showing good specificity and sensitivity. Both showed much higher validity than the AAPD CRA form.

Even with these limited published clinical comparisons the question remains as to which tools are better into the future for CRA. We concluded that it would be useful to compare the above four CRA tools to demonstrate similarities and differences among them. These comparisons are described in the next section.

## Comparison of Four CRA Methods

The age groups used in these comparisons are 0–6 years and 6 years through adult to embrace the slightly different grouping in each of the above-mentioned four CRAs (Cariogram, CAMBRA, ADA, AAPD), some previously using 0–5 years and others 0–6 years. In the case of a 6-year-old child it is the decision of the dental care provider whether to use the 0–6 year CRA tool or the 6 year through adult CRA tool.

### Definitions of Terminology for CRA

In the present publication, risk factors, protective factors and disease indicators as listed in Tables 2, 3 are defined as follows:

a) **Protective factors** are environmental factors, biological factors or chemical therapy that help to swing the caries balance (Figure 1) to caries lesion prevention or reversal. Examples are fluoride in drinking water, the use of fluoride toothpaste and adequate salivary function.
b) **Risk factors** are environmental or biological factors that contribute to the initiation or progression of caries lesions. They include the pathological factors like acid producing bacteria, visible plaque on the teeth, frequent snacking on fermentable carbohydrates as well as environmental factors such as low health literacy (Figure 1, Tables 2, 3).
c) **Disease indicators** are the clinically observed results of previous and/or ongoing dental caries destruction of the tooth mineral. They do not contribute to the disease, but they are direct indicators of the presence of the disease in the past or at the time of the observation.

In order to directly compare each of the above-listed four CRA tools we assembled two groups of hypothetical patient characteristics, one group of 13 for the 0–6 year age group and another group of 13 for the 6 year through adult age group, each group with caries risk levels ranging from low to very high or extreme. We used the evidence-based clinical findings, risk factors and protective factors that have been used for CAMBRA CRA in previous clinical validation studies described above [26, 55] and listed in Tables 2–**5**. The CAMBRA CRA also adds a category of “very high” for the 0–6 year age group and includes “extreme” for the 6 year through adult age group. Examples of these two categories are also included in the following comparisons.

#### Comparison of Four CRA Methods for the Age Group 0–6 Years

Table 4 presents the 13 examples for the age group 0–6 years with CRA levels ranging from low to very high according to the CAMBRA method. These hypothetical examples were chosen to mirror typical clinical examples that ranged from low to very high caries risk, with a variety of combinations of each of the observations and factors listed in Table 2. Obviously many more examples could be included, but the 13 examples chosen cover the more common occurrences.

**Table 4:**
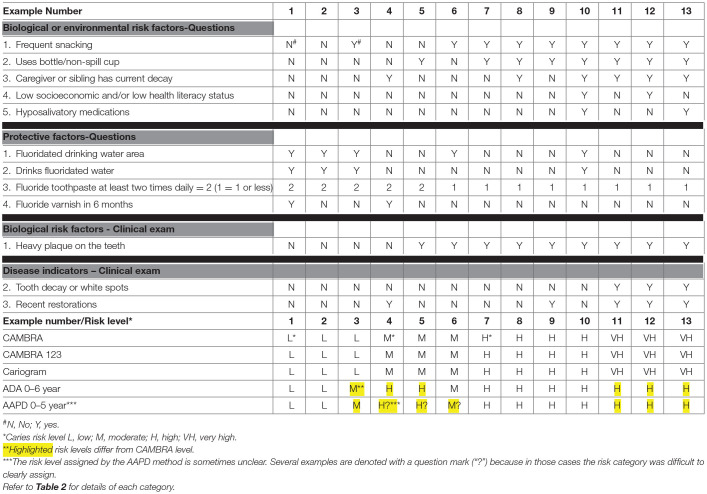
Comparison of 0–6 year age group CAMBRA, CAMBRA 123, Cariogram, ADA and AAPD CRA forms using hypothetical patient example cases.

A further modification of the caries balance method for CAMBRA was added in this work as a potential simple quantitative guide for the dental care provider. We have called this the “CAMBRA 123” method. If it compares favorably with the caries risk levels determined by CAMBRA and Cariogram such an easy numerical system may be a better, more objective guide than working through instructions and visualizing the caries balance. We determined the CAMBRA 1, 2, 3 weightings by experiment using our hypothetical examples (Tables 4, 5). Any yes for an answer to the protective factors is allocated a score of −1. A yes answer for the biological or environmental risk factors is allocated a score of +2 and for clinical disease indicators a score of +3. In order to do the risk assessment, the three columns are added, and the arithmetic balance determined produces a negative or positive number. From the range chart in Table 2 (part 2) the possible caries risk level is produced using this “CAMBRA 123 method.” Then the care provider makes a final decision on the caries risk level by weighing all the factors, information about the patient and her/his clinical judgment. Each of the 13 examples illustrates this process.

**Table 5:**
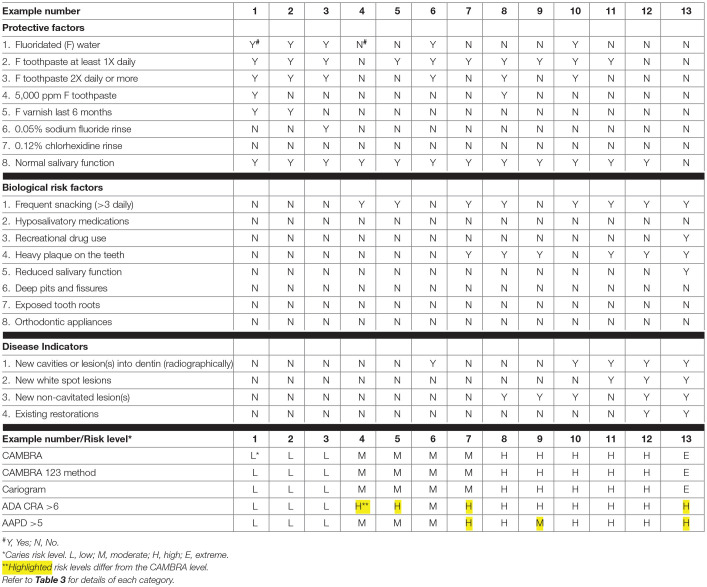
Comparison of Ages 6 year through adult CAMBRA, Cariogram, ADA and AAPD CRA forms using hypothetical patient example cases.

The risk level for the Cariogram CRA method was determined for each of the 13 examples using the internet Cariogram program [33]. The risk level for each example for the ADA and AAPD CRA methods was assessed according to the published instructions [19–21].

### Results of the Comparison of Four CRA Methods for the Age Group 0–6 Years

Table 4 summarizes the caries risk levels obtained by using the above four CRA methods for the 13 examples for the age group 0–6 years. The end result for each of the methods is given at the bottom of Table 4. Results for each method for each example were compared to the CAMBRA method.

1. The Cariogram CRA method gave the same caries risk level result for each of these 13 examples as the CAMBRA CRA in this age group.
2. The CAMBRA 123 CRA method gave the same caries risk level result for each of the 13 example cases as the Cariogram algorithm and CAMBRA in this age group.
3. The ADA 0**–**6 form gave a reasonable match to the CAMBRA and Cariogram caries risk level results. However, one of the three cases that were rated low risk by CAMBRA (example #3) was rated moderate by the ADA CRA. Further, two of the three cases rated moderate risk by CAMBRA (examples #5, #6) were rated as having a high risk by the ADA CRA. The “very high” category does not exist in the ADA CRA.
4. The AAPD 0**–**5 form gave somewhat of a match to the CAMBRA results and uses most of the same protective and risk categories. However, they are combined in a very different way and do not use the caries balance concept. One of the three cases rated as low risk by CAMBRA (example #3) was rated moderate by the AAPD CRA. Further, two of the three cases rated moderate by CAMBRA and Cariogram (examples #5, #6) were rated as high caries risk by the AAPD CRA. The “very high” category does not exist in the AAPD CRA. Several examples are denoted with a question mark (“?”) because in those cases it is not clear what the category should be.

#### Comparison of Four CRA Methods for the Age Group 6 Years-Adult

The rationale for our comparison of the four CRA tools for the age group 6 years through adult was the same as that for the 0–6 year age group, as described fully above. Similarly, 13 hypothetical patient examples were constructed as described above for the 0–6 year age group and used as shown in Tables 3, 5. The same rationale was used for the choice of these examples, except that they were appropriate for the older age group CRA tools. The examples were for patients with caries risk levels ranging from low to extreme according to the previously published CAMBRA CRA tool [24, 28].

As described above for the 0–6 year age group, a further modification of the caries balance method for CAMBRA was added in this work as a simple quantitative guide to assist the dental care provider in determining caries risk. The method is summarized in Table 3, part 2. Again, we call this the “CAMBRA 123 method” for ages 6 years through adult. As described above such a numerical system may be a better guide for the clinician than simply visualizing the caries balance. As for ages 0–6 years any yes for an answer to the protective factors is allocated a score of −1. A yes answer for the biological or environmental risk factors is allocated a score of +2 and for clinical disease indicators a score of +3. In order to do the risk assessment, the three columns are added and the arithmetic balance is determined as a negative or positive number. From the chart in Table 3 (part 2) the possible caries risk level is produced using this “CAMBRA 123 method.” Then the care provider makes a decision on the cares risk level by weighing all the factors and information about the patient. Each of the 13 examples in Table 5 illustrates this process.

The risk level for the Cariogram CRA method was established for each of the 13 examples using the internet Cariogram program [33]. The risk levels for each example for the ADA and AAPD CRA methods was assigned according to the published instructions [20, 21].

### Results of the Comparison of Four CRA Methods for the Age Group 6 Years Through Adult

The determined caries risk level for each of the tools is given at the bottom of Table 5. Assigned risk level results for each tool for each example were compared to the CAMBRA CRA.

1. The Cariogram CRA tool gave the same caries risk level result for each of the 13 examples as the CAMBRA CRA.
2. The CAMBRA 123 CRA method gave the same caries risk level result as CAMBRA and the Cariogram CRA tool for all 13 of these examples.
3. The ADA 6 year through adult CRA tool gave several different results from CAMBRA and Cariogram methods. Examples #4, #5, and #7 were judged as high risk instead of moderate caries risk. The “extreme” category does not exist in the ADA CRA (example #13).
4. The AAPD “greater than 5-year tool” classified a higher risk level in example #7 and a lower risk level in example #9 than CAMBRA and Cariogram. The “extreme” category does not exist in the AAPD CRA as illustrated in example 13.

## Discussion and Conclusions

The management of caries based on individual risk has been recognized as a step forward to achieve better outcomes of oral health [33, 43, 44, 67–70]. Although risk assessment for disease in any field of medicine is an imprecise task, it can assist considerably in targeting those in most need of additional therapy specific to their needs. Using a risk-assessment form that can allow clinicians to assess the impact of their preventive and management strategies over time is imperative to effectively guide patients toward health. Using a method that has sensitive, specific and reproducible results, is as important as leaving some room for the clinicians to use their judgement to compensate for individual/family/social situations that may push the patient to a higher or lower risk category. This is especially important in young children, where their oral health improvement depends on the choices of their parents and their adherence to recommendations for behavior change.

We have provided a listing of numerous CRA tools available and then compared four in detail, namely Cariogram, CAMBRA, ADA CRAs and the AAPD CRAs. The comparison of the Cariogram method and the CAMBRA CRAs in the present paper produced equivalent results for both age groups in the panel of hypothetical patient examples constructed using patient and clinical factors included in the CAMBRA risk assessment forms. The results for the 0–6 year group are in agreement with the published clinical comparison [67] by Agouropoulus et al. that concluded that Cariogram and CAMBRA were equivalent methods and that each may be used as guidelines for the health care provider in determining caries risk in this age group. In that clinical study both Cariogram CRA and CAMBRA CRA showed much higher validity than the AAPD CRA form.

The CAMBRA 123 quantitative CRA method introduced in this publication when used and compared as presented above (Tables 2–5) gave equivalent results to Cariogram and CAMBRA in both age groups for all of the theoretical case examples. This new quantitative method for utilizing the caries balance to assist in determining caries risk may be a useful, fast and easy guideline for the health care provider in determining caries risk for the 6 year through adult age group as well as the 0–6 year age group. The CAMBRA 123 method is more objective than the judgment-based CAMBRA method that has been used for over 15 years in clinical practice and that has been validated in clinical studies. Although the CAMBRA 123 method performed well in these comparisons it is a derived method and has not, however, been tested or validated by clinical outcomes studies.

In the comparisons presented in this publication the ADA and the AAPD tools differed from Cariogram and CAMBRA tools in the assignment of caries risk levels in several examples in each age group. Although the clinical observations, risk factor and protective factor categories of the ADA and AAPD tools are similar to CAMBRA they are combined in a very different way and do not use the caries balance concept. The ADA CRAs tend to rate the cases at higher risk categories than the CAMBRA system for both age groups. The ADA 0–6-year CRA is more likely to categorize a patient as high risk when the category of “bottle other than water at bedtime” is present. The AAPD CRAs also tend to assign higher caries risk categories than CAMBRA. For ADA and AAPD CRAs the preventive factors are given less weighting than they are given in the CAMBRA CRAs. In the AAPD 0–5-year CRA form much is left to the judgment of the provider and it is sometimes unclear what risk level might best be assigned.

It is important to use a CRA form or electronic tool not only as a checklist to determine caries risk but also to use the details to create a caries management plan [43, 44, 67, 71, 72]. The outcomes of preventive and restorative treatment, whether surgical or non-surgical, greatly depend on the patient's understanding of their individual risk factors and their behavior changes that will allow them to tip the caries balance toward health. Using a system that combines all the information gathered during risk assessment to empower the patient with knowledge of factors relevant to them personally can be very helpful to lead them to improve their health choices, especially when reinforced periodically. The validity of the Cariogram and CAMBRA tools is supported by clinical outcomes evidence [14, 54, 55, 67]. Health care providers can expect to obtain similar risk classifications from the previously published CAMBRA and Cariogram CRAs in determining caries risk for both age groups.

In conclusion, the present paper offers a review of the successful CAMBRA CRA tool that can be the foundation for caries management systems for the age groups 0–6 years and 6 years through adult. Both the Cariogram and the CAMBRA CRA methods are equally useful for identifying the future risk of dental caries. CAMBRA 123 shows promise as an easy to use quantitative method for CRA in clinical practice. The health care providers will be the ones to decide which CRA method will allow them to establish individualized, successful caries management therapies and how to combine these for the best care of their patients.

## Author Contributions

JF, YC, PA, BC, SD, PR, LZ, and FR-G contributed to the planning, writing, scientific and clinical content, reviewing, and editing of this document. All authors contributed to the article and approved the submitted version.

## Conflict of Interest

The authors declare that the research was conducted in the absence of any commercial or financial relationships that could be construed as a potential conflict of interest.
